# Cultivation of the Acidophilic Microalgae *Galdieria phlegrea* with Wastewater: Process Yields

**DOI:** 10.3390/ijerph18052291

**Published:** 2021-02-26

**Authors:** Maria Rosa di Cicco, Maria Palmieri, Simona Altieri, Claudia Ciniglia, Carmine Lubritto

**Affiliations:** 1 Department of Environmental, Biological and Pharmaceutical Sciences and Technologies, University of Campania “Luigi Vanvitelli”, Via Vivaldi 43, 81100 Caserta, Italy; maria.palmieri@unicampania.it (M.P.); simona.altieri@unicampania.it (S.A.); claudia.ciniglia@unicampania.it (C.C.); carmine.lubritto@unicampania.it (C.L.); 2 INFN—Sezione di Napoli, Complesso Universitario di Monte S, 80126 Napoli, Italy

**Keywords:** bioremediation, *Galdieria*, biomass yield, isotopic ratio, lipid, nutrient removal, urban wastewater

## Abstract

Algal based wastewater treatment offers the opportunity to recover, in the form of biomass, the nutrients and internal chemical energy of wastewater. Recently, there has been a growing interest in the use of extremophilic microalgae, as they can easily adapt to difficult and often pollutant-rich environments. The thermo-acidophilic microalga *Galdieria phlegrea* is a species of recent discovery and great metabolic versatility, but it has still been poorly studied. Here, *G. phlegrea* was cultivated using raw municipal wastewater in 1 L Erlenmeyer flasks with 700 mL working volume at 37 °C for up to nine days. During the cultivation phase, biomass growth, phycocyanin content, ammonium and phosphate removal from the wastewater, lipid fraction, total carbon and nitrogen in the biomass, and variation in δ^13^C and δ^15^N isotopic ratios (a novel analytical contribution in these experiments) were monitored. Results indicated that *G. phlegrea* was able to grow in raw effluent, where it removed more than 50% ammonium and 20% phosphate in 24 h; total lipid content was in the range of 11–22%, while average C-N content was of 45% and 6%, respectively; isotopic analyses proved to be a useful support in identifying C and N metabolic pathways from effluent to biomass. Overall, *G. phlegrea* showed consistent performance with similar *Cyanidiophyceae* and is a potentially viable candidate for municipal wastewater valorization from a circular economy perspective.

## 1. Introduction

Urban wastewaters are mainly rich in different forms of carbon (C), nitrogen (N) and phosphorous (P), to be removed prior to discharge into receiving water bodies. Despite the technological advances achieved in recent years, a large number of wastewater treatment facilities (WWTFs) still entrust the removal of the pollutant load to activated sludge technologies, which are well established but also energy-intensive and resource-wasting multistep processes [1,2]. Such systems oxidize the organic carbon to CO_2_ through heterotrophic bacteria commonly flourishing in the sewage, which use oxygen as a terminal electron acceptor in their metabolic chain [3]. Unfortunately, since the organic carbon in urban wastewater is not stoichiometrically sufficient for such heterotrophic bacteria to balance all the N and P as much efficiently as possible [3], a disadvantage of this process is that the use of activated sludge alone is inadequate to comply with the discharge standards for all nutrients [4], thus requiring additional energivorous treatments (tertiary treatments), all leading to a great waste of resources. For example, one of the most common solutions is biological nutrient removal (BNR) driven by ammonia oxidizing bacteria, which captures P into biosolids and the converts ammoniacal nitrogen into N_2_ gas through the alternation of nitrification/denitrification [5]. BNR systems can account for 60–80% of the total consumption of a WWTF [6] and, since urban wastewater has an energy content of 6.3–7.6 kJ L^−1^ [7], which exceeds the energy used to treat such wastewater [8], reclaiming this energy would be an interesting opportunity. Moreover, sewage is rich in N and P, which could be recovered for the market as agricultural fertilizer [9,10]. Another strategy to enhance wastewater treatment relies on the possibility of replacing anaerobic metabolism with different metabolic processes to avoid the lack of dissolved oxygen in the tanks [11]. A more interesting study proposes the use, in the activated sludge process, of reduced sulfur compounds (i.e., sulfide, thiosulphate, elemental sulfur) to serve as additional electron donors to drive the sulfur cycle reactions that lead to the reduction of nitrate and nitrite to dinitrogen gas (autotrophic oxidative sulfur denitrification) [12].

An entirely different approach that has been gaining great interest in recent years is the implementation of algal and mixed algal/bacterial systems as a sustainable alternative for urban wastewater treatment and energy production [13,14,15]. Being metabolically flexible organisms capable of autotrophy, mixotrophy or heterotrophy, microalgae can potentially constitute beneficial and promising bio-systems for the treatment of different wastewater streams [16]. According to a biorefinery concept and policies towards bio-based circular economies [17,18], the idea is to use wastewater as a sustainable feedstock for microalgal biomass, from which bioproducts can be obtained [19], such as: protein and storage carbohydrates (e.g., glycogen) [20], pigments [19,21], lipids [22], biofertilizers [13] and biocrude-biochar via hydrothermal liquefaction [23].

However, adopting microalgae by integrating them into pre-existing WWTFs is not only aimed at generating the above-mentioned products. Indeed, the use of these organisms can truly support the sewage purification process. The rationale is that algal microorganisms use solar energy to supply oxygen through photosynthesis; O_2_ is then employed for the oxidation of organic carbon to CO_2_, which is, in turn, necessary for the photosynthetic production of biomass [24]. The additional oxygen supplied by microalgae could reduce the amount of gas that must be supplied materially from outside by direct blowing or mechanical mixing; this is a very important contribution, because it helps to reduce total energy costs and improve system performance [16]. Unlike conventional treatments, this system would make it possible to produce a biomass potentially richer in energy, deriving both from the internal energy of the wastewater and from solar radiation.

At present, experimental studies on the use of microalgae for wastewater treatment favored conventional microalgae and cyanobacteria and were characterized by a tendency to accumulate high levels of lipids and storage starch [16]; examples of these organisms are *Arthrospira* sp. [25], *Chlorella* sp. [26], *Scenedesmus* sp. [27] and *Spirulina* sp. [28]. Several factors affect the selection of the strain to be used. For example, to maximize the extraction of compounds of high biotechnological value such as phycocyanin or glycogen, the latter being a potential food supplement in the nutraceutical sector [29], preference should be given to a strain that exhibits a higher yield of such compounds, while being able to thrive in urban wastewater. Nevertheless, particular attention should be paid to the selection of an algal strain which is able to properly reduce not only the pollutant load of the sewage, but also the community of pathogens naturally living in the wastewater. Particularly for *E. coli*, it has recently been shown that activated sludge treatment indeed promotes an increase in its antimicrobial resistance genes, thus posing a threat to public health [30].

Within the context of algal wastewater treatment, one of the most interesting and recent approaches is the one involving *Galdieria*, a polyextremophilic red microalga belonging to the class *Cyanidiophyceae* [31,32]. *Galdieria* thrives in geothermal volcanic soils with temperatures reaching 50 °C and high sulphuric concentrations [33,34,35]. *Galdieria* possesses an extraordinary metabolic versatility, thanks to the presence of a large number of membrane transporters and enzymes of carbohydrate metabolism. These microalgae can easily pass from a photoautotrophic growth regime to a mixo-/heterotrophic growth regime on more than 50 different carbon sources, such as glucose, glycerol and food waste rich in organic matrix [36]. It has a significant flexibility of adaptation to pH up to 5.5, despite its natural conditions characterized by a pH below 2 [35]. *Galdieria* has high potential in biotechnological applications and it is a reliable means for applications in bio-based remediation processes because of its natural ability to cope with and neutralize biohazardous heavy metals [37] typically present in volcanic areas and acid mines, thanks to the presence of a wide variety of carriers [29]. Moreover, being able to tolerate temperatures up to 56 °C [29], these microalgae can be cultivated in closed photobioreactors exposed to full sunlight and high diurnal temperatures without energy-consuming cooling technologies [24], thus achieving a reduction in energy costs of the system. Furthermore, these organisms draw a lot of interest because they exhibit high phycocyanin levels, ≈1–2 orders more than other species, while being able to accumulate under appropriate conditions up to 50% of glycogen, the latter having a lower molecular weight and a more branched chain compared to the one produced by other organisms [29].

As these features generate high interest from the biotechnological sector (e.g., glycogen could be used instead of starch), it is important to look at alternative and economic strategies for the cultivation of these algal strains and urban wastewater represents a sustainable opportunity. The research on the use of *Galdieria* genus for wastewater treatment is very recent. Up to now, the most comprehensive and advanced experience of *Galdieria* application comes from the Las Cruces WWTF (New Mexico, US), where a pilot system denominated “POWER” (first mentioned in Selvaratnam, et al. [38]) has been implemented for the cultivation of *Galdieria sulphuraria* species in primary settled urban wastewater collected on-site, followed by production of biocrude and biochar through a well-established hydrothermal liquefaction process and the recirculation of the nutrient-rich residual aqueous fraction as a feed for algal culture [14,39,40,41,42,43]. Since the research has been performed in New Mexico, the idea was to implement a system at the local WWTF which could be optimized for hot and arid regions, where high diurnal temperatures are typical and water scarcity is a serious concern [44].

Using “POWER” bibliographic background as the main benchmark, this paper provides the results obtained from the experimentation of the strain *Galdieria phlegrea* ACUF 784.3. *G. phlegrea* is a recently discovered *Galdieria* species [45], still poorly investigated in the scientific community, and an application with urban wastewater is still missing. *G. phlegrea* has proved to not be a strictly thermophile [46], so it could represent an alternative to *G. sulphuraria* in less extreme climates. Moreover, the metabolic versatility of *Galdieria* genus makes its species promising candidates for treating high chemical oxygen demand-loaded streams [16]. Related to this, the urban wastewaters employed in these experiments were collected at a large WWTF located in southern Italy, which are negatively affected by dilution of organic load and are characterized by a high COD/BOD_5_ ratio [47,48,49]. Experimentation consisted of growing the new *G. phlegrea* strain in primary settled urban wastewater, whereby the main process parameters were monitored over time, including: biomass production, ability to reduce NH_4_^+^ and PO_4_^3−^ as pollutant load, phycocyanin and total lipid content. The results provide a higher temporal resolution level than what is available in most analogous works because the parameters were monitored at very short time intervals (<24 h). A further innovation was the use of EA-IRMS, which is not only used to accurately characterize C and N total content in *Galdieria* species, but mainly to study the variation of the stable isotope ratios δ^13^C and δ^15^N during the contact between microalgae and growth medium. This analytical tool was confirmed to be a valid support in understanding and describing the metabolic processes immediately undertaken by the organism when it comes into contact with a new substrate. Stable isotope analysis is a novelty in this type of experiment, whereas similar examples concerning the relationship between microalgae and urban wastewater are not available in previous literature.

This study not only provides valuable data for international discussion on a microorganism that today is not fully known, but also, by bringing together analytical techniques from multiple research areas, it highlights the usefulness of pursuing interdisciplinary approaches in the study of innovative and sustainable processes.

## 2. Materials and Methods

### 2.1. Algal Culture and Growth Media

**Figure 1:**
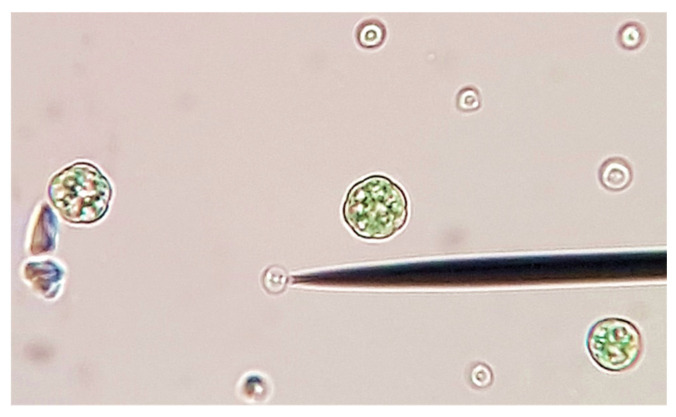
Spore formation in *Galdieria phlegrea* ACUF 784.3 (original photo).

*Galdieria phlegrea* ACUF 784.3 (Figure 1) was provided by the Algal Collection of University of Naples (www.acuf.net (accessed on 11 June 2019)). The algal strain was firstly isolated by streaking it across agar plates, and colonies were inoculated in Allen medium pH 2.5 [50] and cultivated at 37 °C under continuous fluorescent illumination of 45 µmol photons·m^−2^·s^−1^.

Allen medium was composed as follows [50]: (NH_4_)_2_SO_4_ 0.01 M; MgSO_4_*7H_2_O 0.001 M; CaCl_2_*2H_2_O 0.0005 M; KH_2_PO_4_ 0.002 M; Fe 4 mg L^−1^; Mn 0.5 mg L^−1^; B 0.5 mg L^−1^; Zn 0.05 mg L^−1^; Cu 0.02 mg L^−1^; Mo 0.01 mg L^−1^; V 0.01 mg L^−1^; pH adjusted to 2.5 by means of H_2_SO_4_ and pH-meter. During the preparation stage, in order to promote mixotrophic growth and higher biomass productivity, the medium was also enriched with sucrose (2.0–2.5 g L^−1^).

The cell concentration of the stock culture was assessed through microscope cell counting via hemocytometer, which is a tool specifically designed to count high concentrations of organisms of small size (<30 µm) using a microscope. The hemocytometer is made from a thick glass on which perpendicular lines are engraved to form a grid, which can accommodate a known volume of culture, in our case 0.0009 mL [51]. After determining the cell concentration with hemocytometer, 5 dilutions were made from the stock culture and, for each one of these dilution points, the optical density was measured at 750 nm (OD_750_) by spectrophotometer. Each dilution point was prepared in duplicate and, for each sample, spectrophotometric measurements were made in triplicate, for a total of 30 observations and an r^2^ of 0.994. Following this procedure, a calibration curve was drawn for the algal strain, in which each value of OD_750_ corresponded to a cell concentration. Once the calibration was completed, using the method of least squares the volume ***x*** was derived, which had to be harvested and centrifuged from the stock culture to be inoculated in the samples of the experiments. The spectrophotometer used for these operations was a Shimadzu UVmini-1240, for single cuvettes with an optical path of 1 cm.

The urban wastewater employed in the experiments (**W**) were collected at the inlet of the oxidation tank, so as to have a wastewater without suspended solids and grease, and which had not yet undergone any biological treatment. After the sampling, the wastewater was left to decant 1 day at 4 °C and then brought to a pH of 2.5 using H_2_SO_4_ and a pH-meter. For the control of the experiment, we decided to use the modified Allen medium (**A**) described above, at pH 2.5 and without addition of sucrose, to promote only autotrophic metabolism.

### 2.2. Experimental Setup

A volume ***x*** of *G. phlegrea* was harvested from the stock culture and centrifuged at 4000 rpm for 4′ at room temperature. The sedimented biomass was washed twice with the target medium (W or A) and it was finally suspended in a volume of 700 mL in 1 L Erlenmeyer flasks, to reach a final cell concentration of about 10^6^ cells mL^−1^ (OD_750nm_ ≈ 0.8). Three biological replicates were prepared for each of the two conditions W and A, and each analytical measure was performed in triplicate. 120 mL of the cultures were collected from each Erlenmeyer flask at the start (0 h) and after 0.5 h, 3 h, 6 h and 24 h, and used for the assessment of the following physiological parameters: (i) biomass as *Ash Free Dry Weight* (AFDW) and its total phycocyanin content; (ii) cell counting; (iii) ammonium and phosphate removal; (iv) total lipids in the biomass; (v) total content of C and N in the biomass and the corresponding isotopic ratios δ^13^ C and δ^15^N.

Optical density and cell counting with hemocytometer were monitored in parallel every 3 days until day 9; the ammonium concentration was evaluated up to day 3. Table 1 shows the time pattern of the samples taken from each biological replicate and the corresponding evaluated parameter.

#### 2.2.1. Ammonium and Phosphate

Ammonium and phosphate variations were quantified spectrophotometrically by following the method described by Hernández-López and Vargas-Albores [52].

This method was chosen because, operating on very small volumes in microplate (250 μL in each well), it allows the simultaneous analysis of the entire panel of collected samples, thereby reducing the uncertainty related to multiple analyses.

In particular, for the determination of ammonium, 250 μL of the samples were mixed in a microplate with 3 different reagents: 20 μL of 10% phenol solution (in 95% ethanol), 20 μL of 0.5% sodium nitroprussiate and 30 μL of a reactive solution composed of 10 mL alkaline solution (10 g sodium citrate + 0.5 g sodium hydroxide in 50 mL deionized water) with 2.5 mL commercial sodium hypochlorite. The mixture was incubated for 60′ at room temperature in the dark (to prevent any radiation interference) and the optical density was recorded at 655 nm. Ammonium sulphate (200 μM) was used as a standard to prepare a calibration curve in the range 1.6–50 μM.

To determine phosphate content, 250 μL of the samples were placed in a microplate containing 30 μL of reactive solution in each well (0.6% ammonium heptamolybdate, 12.75% sulphuric acid, 1.08% ascorbic acid, and 0.0163% each of antimony and potassium tartrate) and incubated for 10 min at room temperature. The absorbance was determined at 655 nm. A 100 μM KH_2_PO_4_ solution was used to prepare a standard curve ranging from 0.4 to 60 μM.

#### 2.2.2. Biomass Concentration and Phycocyanin Content

During the experiment, the biomass concentration was measured both spectrophotometrically and by cell counting using a hemocytometer. Spectrophotometric assessment of the biomass content was carried out by measuring the OD_750_ of the samples, and the obtained value was converted to mg L^−1^ using the relation described from Selvaratnam, Pegallapati, Montelya, Rodriguez, Nirmalakhandan, Van Voorhies and Lammers [44] for *G. sulphuraria*:

AFDW [g L^−1^] = 0.54 × OD_750_ + 0.023; [n = 12; r^2^ = 0.997]. (1)

At the same time, the total phycocyanin content was also evaluated spectrophotometrically by measuring the absorbance of the algal suspension at 624 and 652 nm and then applying the formula [53,54]:

Phycocyanin [mg L^−1^] = [(OD_624_ − (OD_652_ − 0.474))/5.34] × 1000. (2)

#### 2.2.3. Lipid Content

Since *G. phlegrea* is a thermophilic microalga with a very strong external cell membrane, the lipids contained in the biomass were extracted with the n-hexane Soxhlet method assisted by ultrasonication and freeze-drying of the biomass, according to the procedure reported by Onay, et al. [55] for microalgae resistant to high temperatures. Specifically, for each sample, 100 mL of algal culture were pre-treated with ultrasonic bath (Elmasonic S10) in ice water at 37 kHz for 30′. Then, the culture was centrifuged at 4000 rpm for 5′ at 4 °C to isolate the disrupted biomass, which was then freeze-dried. After this pre-treatment, each lyophilized biomass sample was placed into a filter bag (F57, Ankom Technology), which was sealed and labelled in binary code. The filters thus obtained were inserted into the Soxhlet to initialize the 24 h extraction cycle with n-hexane [55]. At the end of the cycle, filter bags were completely dried in a laboratory oven at 40 °C for 24 h. Then, the amount of lipid extracted from the biomass was derived with a gravimetric method by difference in weight between the initial biomass and the final one. For all weighing, a high-precision balance with a sensitivity of 2 μg was used. To assess the initial lipid content in the biomass, a sample of the stock culture was collected.

#### 2.2.4. C and N Content and Evaluation of δ^13^C and δ^15^N

Since two isotopes of the same element differ in mass number, they have different physical properties and their relative abundances do not retain a rigid structure in nature, but variations occur due to chemical, physical or biological processes [56]. The relative distribution of heavier and lighter isotopes between two coexisting phases in a natural system is called isotope fractionation [57]. Metabolic activity in organisms, or the interaction of volatile elements with aqueous and crustal material, are all processes that induce predictable enrichment or depletion in a specific isotope [58]. For this reason, there are distinct isotope fractionation models that allow the interpretation of the prevailing processes involved, and these patterns can be used not only in the description of past events, but they are also widely used in the observation and analysis of real time phenomena [59]. The isotopic composition of elements in matrices of environmental interest is generally expressed in terms of delta notation (δ). In practice, this parameter expresses how many parts per thousand (‰) of the considered isotope ratio deviate from the same ratio of a reference material with a known isotopic composition. A value of δ > 0 indicates that the sample is enriched in heavier isotopes with respect to the standard.

With reference to the research discussed in this study, the isotopic analyses were carried out because they are an analytical tool that provides great support in the identification and understanding of metabolic processes taking place. To prepare the samples for the analyses, algal biomass isolated from 20 mL samples was freeze-dried at −80 °C without pre-treatment. In order to characterize the baseline of these parameters in the biomass, a sample from the stock culture was also collected and freeze-dried. After that, ≈800 μg of the dried biomass was weighed and encapsulated in 4 × 6 mm tin capsules (Tin capsules for solids, Santis analytical). These samples were then analyzed with IRMS Isotopic Ratio Mass Spectrometer (Delta V Advantage—Thermo Fisher, Waltham, MA, USA) coupled to an EA Elemental Analyzer (1112 Series—Thermo Fisher, Waltham, MA, USA), for the simultaneous measurement of C and N contained in the biomass (%) and their relative isotopic ratios δ^13^C and δ^15^N (‰). The reference standards are the Vienna PDB for the ratio ^13^C/^12^C (= δ^13^C) [60] and the Air for the ratio ^15^N/^14^N (= δ^15^N) [61].

#### 2.2.5. Statistical Analysis

The experiments were performed by preparing 3 biological replicates in Erlenmeyer flasks for each condition (A_1–3_, W_1–3_) and, from each individual flask, the collected samples were tested in triple technical replicate for every parameter to be measured. The significance of the growth/decrease of the parameters was evaluated by means of paired-sample *t*-test with level of significance *p* = 0.05. As a further check, a Tukey’s range test was also carried out on the data sets. The graphical representations were prepared using Microsoft Office Excel, while OriginPro 2018 software (©OriginLab Corporation) was used for statistical analysis.

## 3. Results and Discussion

### 3.1. Biomass Growth and Phycocyanin Content

**Figure 2:**
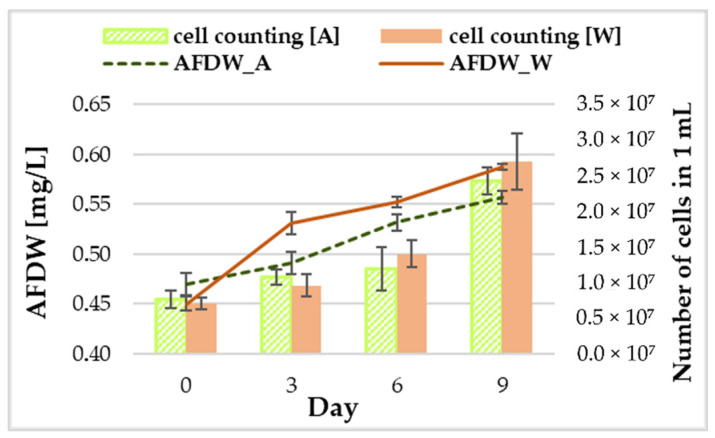
Comparison between Ash Free Dry Weight (AFDW) and cellular concentration evaluated via cell counting. Results are reported as means ± standard deviation (*n* = 9).

*G. phlegrea* ACUF 784.3 was able to grow on wastewaters (W) as well as in the control (A), consistently with what is reported in the relevant literature for similar experiments with *G. sulphuraria* [44]. In particular, our results (Figure 2) suggest that the growth in wastewater is higher than in the experimental control, especially in the initial growth stage. In numbers, starting from an inoculum of about 0.45 g L^−1^, corresponding to an optical density at 750 nm of about 0.8 and a number of cells in the order of magnitude of 10^6^ cells mL^−1^, growth peaked at 0.588 g L^−1^ in wastewater and at 0.557 g L^−1^ in the control after nine days in batch mode (*p* < 0.05). Therefore, the sample that at the end of the experiment showed the highest density (by 10%) was the one grown in wastewater, similarly to what also demonstrated by Selvaratnam, Pegallapati, Montelya, Rodriguez, Nirmalakhandan, Van Voorhies and Lammers [44] with *G. sulphuraria*.

Discrepancies between our tests and those reported in literature might be ascribable both to a different culture system and to the highest pollutant load of the urban primary settled wastewater [44]. In fact, NH_3_-N and PO_4_^3^ concentrations in the wastewater employed within the POWER project were typically of about 40 mg L^−1^ and 10 mg L^−1^, respectively [38], much higher than the value reported for the wastewater from the city of Salerno where both ammonium and total phosphorus had an average value of about 5 mg L^−1^ [49]; this was mainly due to the fact that the wastewater treatment facility was affected by a large amount of undesired inflows/infiltrations diluting the wastewater [47,48]. The hypothesis is that the scarcity of nutrients would not allow a growth at the levels reached in New Mexico, where sewages were much more concentrated in eutrophic elements.

Interesting evidence emerged from the comparison between ash free dry weight obtained through spectrophotometric measurements and microscope cell counting of the samples (Figure 2). Results showed that over time the increase in optical density did not necessarily correspond to an increase in the number of cells (Figure 2). This may depend largely on two dynamics. During adaptation and growth, cells spend a period of time increasing their volume: in this case, with the same number of cells, the optical density (and so the AFDW) is higher because the cells are bigger in size. When microalgae reproduce, they generate cells of very small size: in this case, with the same optical density, the microscope cell counting will result in a larger number of cells with smaller size.

Finally, with regard to the total content of phycocyanin, no significant variations were appreciated over time and its value was about constant in all the experiments around an average value of 94 ± 6 mg L^−1^.

### 3.2. Variation of NH_4_^+^ and PO_4_^3−^

**Figure 3:**
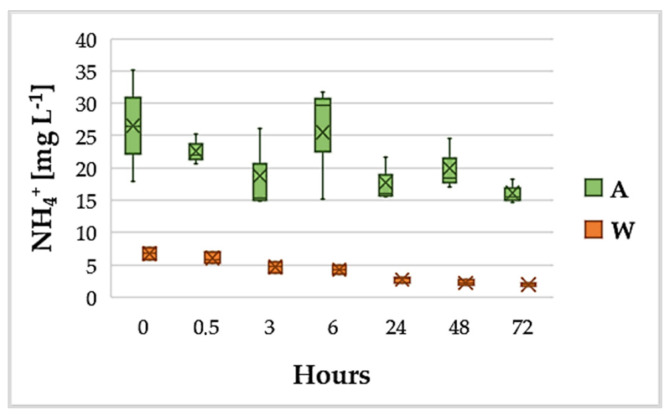
Variation of NH4^+^ in the wastewater (W) and in the standard growth medium (A).

Figure 3 shows the trend of NH_4_^+^ during 72 h. The removal efficiency was more than 50% after 24 h with primary settled wastewater containing about 6 mg L^−1^ of NH_4_-N at the start of the experiment; after 72 h, ammonium removal exceeded 70%.

The removal efficiencies obtained in this experiment were well above the value obtained by Selvaratnam, Pegallapati, Montelya, Rodriguez, Nirmalakhandan, Van Voorhies and Lammers [44] in batch mode, when *G. sulphuraria* removed 25% of NH_4_^+^ and 10% of PO_4_^3−^ after 24 h. It is already demonstrated that in Cyanidiales the ammonium uptake depends both on the number of cells in suspension and on the concentration of ammonium in the medium [62]. This implies that the more abundant the ammonium, the faster the uptake will be; on the other hand, with the same ammonium in the medium, the more algae cells are present in the medium and the faster the process will be [62,63,64]. In our work, wastewaters had an initial ammonium concentration of about 6 mg L^−1^ and the algal biomass inoculated was about 0.4 g L^−1^. In Selvaratnam, Pegallapati, Montelya, Rodriguez, Nirmalakhandan, Van Voorhies and Lammers [44], an initial biomass of about 0.1 g L^−1^ was inoculated in wastewater containing about 40 mg L^−1^ of ammonium. What can be assumed is that in the first case a greater quantity of algae is able to abate more quickly a more discrete amount of ammonium.

**Figure 4:**
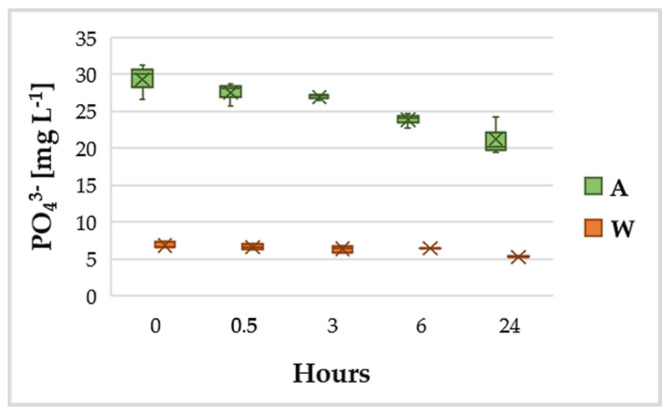
Variation of PO_4_^3−^ in the wastewater (W) and in the standard growth medium (A).

Phosphorus (P) is essential for living cells, including microalgae. It is a component of nucleic acids, structural phospholipids, proteins, sugar phosphates and other metabolites. Several algae assimilate more P than the needed for immediate growth, sometimes forming polyphosphate granules as P deposit. Phosphate removal from wastewater after 24 h was of about 22%, starting from an initial concentration of about 7 mg L^−1^ (Figure 4).

The result obtained for phosphate removal efficiency in wastewater confirmed what was observed for ammonium. Even in this case, in fact, from an initial quantity of phosphate slightly lower than that reported for Las Cruces wastewater (7 vs. 10 mg L^−1^) and inoculating a greater quantity of microorganisms, the value of the removal efficiency is about doubled.

The average ammoniacal nitrogen removal rate attained in raw wastewater during the first 72 h is 3.90 mg L^−1^ d^−1^, with a biomass productivity in the wastewater of 23.18 g of biomass per g of NH_4_-N removed. The value of biomass productivity in wastewater was slightly lower than the average reported in the literature for *G. sulphuraria* from Selvaratnam, Pegallapati, Montelya, Rodriguez, Nirmalakhandan, Van Voorhies and Lammers [44] (27.42 g g^−1^). Higher nitrogen removal rate and biomass productivity values reported in the literature are very often associated with higher initial concentrations of N and P in the growth medium [4,65,66,67,68], as already hinted before. The phosphate removal rate was 1.5 mg L^−1^ d^−1^, 1.25 times the rate of 1.2 mg L^−1^ d^−1^ reported for batch mode experiments with Las Cruces wastewater [44]. The difference observed could be evidence of a greater ability by *G. phlegrea* to capture phosphorus compared to *G. sulphuraria*. To be validated, this hypothesis needs further experiments by switching from batch mode to a continuous system, where removal rates and efficiencies remain nearly constant over time.

### 3.3. Lipid Content

**Figure 5:**
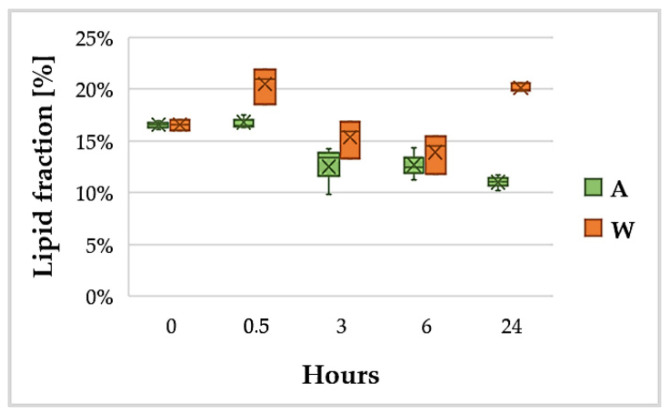
Variation of the lipid content in the biomass grown in wastewater (W) or Allen medium (A). The 8 h of darkness in the climatic chamber occur between 6 h and 24 h.

Using the method described in Section 2.2.3 [55], a total lipid fraction in the range 11–22% was extracted (Figure 5). As can be seen in Figure 5, the pattern of lipid content identified the tendency to a major production of lipids in the raw wastewater, with an evident disparity after 24 h (11% vs. 20%).

Figure 5 shows that, as soon as *G. phlegrea* reached equilibrium with the surrounding medium, lipid content started to decrease in both conditions A and W until the 6th hour. The higher lipid production achieved in W in the first 30′ of contact (0.5 h) was found to be non-significant according to Tukey’s test. Bearing in mind that lipid production is a response to situations of physiological stress [69,70], this initial decrease can be explained by the fact that the abundance of nutrients in the fresh culture media A and W is a favorable condition for the organism, so that lipid production decreases. In the following hours, however, while the lipid content in the biomass grown in autotrophic Allen medium remained stable (non-significant statistical difference), it increased in the biomass grown in wastewater (significant difference). Wastewater from the city of Salerno, despite being strongly diluted [48,49], contains organic carbon, so it can be easily assumed that the microalgae suspended in raw wastewater were able to grow heterotrophically when the light was absent. This condition of light absence in the climatic chamber occurred between the 6th and 24th hour (Figure 5).

In order to explain this response, reference was made to the relevant literature about the amount of lipids extracted in other species of the genus *Galdieria*. Graziani, et al. [71] reported a total concentration of lipids (extracted with a mixture of chloroform:methanol) in *G. sulphuraria* below 2%, with a higher total lipid productivity in autotrophic metabolism rather than heterotrophy; in the same experiment, the content of PUFA (polyunsaturated fatty acids) was higher in heterotrophy and, in general, under stress conditions. López, et al. [72] obtained with *Galdieria* sp. USBA-GBX-832 results similar to Graziani, Schiavo, Nicolai, Buono, Fogliano, Pinto and Pollio [71]; in particular, using a modified version of the Bligh and Dyer extraction method [73], they were able to extract about 3–4% of total lipids in heterotrophy and mixotrophy and 15% in autotrophy. In contrast to the cases just mentioned, but in agreement with our results, Sakurai, et al. [74] obtained with *G. sulphuraria* a higher lipid production in heterotrophy rather than autotrophy using a modified version of the extraction method described in Minoda, et al. [75]. Using GC-MS, Sakurai, Aoki, Ju, Ueda, Nakamura, Fujiwara, Umemura, Tsuzuki and Minoda [74] provided a detailed analysis of the different typologies of lipids constituting the total fraction. More specifically, they observed that the elongation and unsaturation of fatty acids in the endoplasmic reticulum appears to be activated under heterotrophic conditions. However, the mechanisms that induce this process are not yet known. Their observations suggest that this process is influenced more by the cellular metabolic state than by the absence of light and it depends to a large extent on growth conditions [74].

It is important to notice that all these results reported in literature were obtained from experiments in which different *Galdieria* strains, culture media, growth conditions and extraction methods were used. The use of an appropriate extraction method is crucial, especially for algae of the genus *Galdieria*, which have a robust cell wall [71]. For this reason, before using solvents for extraction, it is necessary to ensure good disruption of the biomass to be processed [55]. Since *G. phlegrea* is also a thermophile, for the present research it was decided to use a different extraction method (described in Section 2.2.3) adapted for thermo-resistant microalgae such as those of the *Galdieria* genus, which inherited from Archaea the characteristic of longer hetero-lipids coating the phospholipidic bilayer and preventing its degradation at high temperatures [76]. According to Onay, Sonmez, Oktem and Yucel [55], to improve lipids extraction, it is necessary to apply ultrasonication to the biomass in ice water and then freeze-dry it. Sakurai, Aoki, Ju, Ueda, Nakamura, Fujiwara, Umemura, Tsuzuki and Minoda [74] also performed cold ultrasonication of the biomass, but the cells were first dissolved in methanol. Indeed, by applying in the present research the protocols of pre-treatment and extraction described by Onay, Sonmez, Oktem and Yucel [55], total lipid content extracted was higher, on average, compared to data from previous literature. This result may not only be related to the strain used in the experiments, but it may also indicate that the pre-treatment and extraction methods adopted in this study were more appropriate for the selected microorganism.

### 3.4. C and N Content and Variation of the Isotopic Ratios δ^13^C and δ^15^N

The reactions involving organic carbon through photosynthetic processes determine a decrease of δ^13^C, because during photosynthesis the organic material that is produced becomes enriched in the lighter isotope rather than in the heavy one. As a reference, aquatic plants have a δ^13^C in the range −14/−19 [77].

For nitrogen, naturally abundant in the atmosphere, the first step is its fixation from atmospheric molecular nitrogen to ammonium nitrogen (δ^15^N ≈ 1‰) [77]. The next reactions comprise mineralization, volatilization, nitrification and denitrification, mostly mediated by microorganisms. When referring to the liquid phase, as in the case of the growth media involved in these experiments, the balance between ammonia in solution and in gaseous phase induces an enrichment of the heavier isotope in the solubilized fraction and an increase of δ^15^N for the ammonium ion dissolved in the liquid substrate [77].

**Figure 6:**
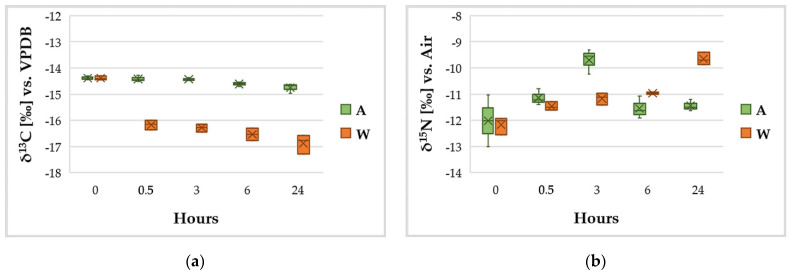
Variation of δ^13^C (**a**) and δ^15^N (**b**) in the biomass grown in wastewater (W) and Allen medium (A).

In these experiments, carbon and nitrogen were analyzed both in terms of total content (%) and isotopic ratios δ^13^C and δ^15^N (Figure 6).

Concerning the total content of C and N, after an initial phase of adaptation to fresh medium, the amounts of these elements did not undergo significant variations in almost all the conditions observed (*p* > 0.05). The only exception was the carbon content in W medium, which varied significantly after 24 h; more specifically, starting from an average C content of 42%, total C amount in biomass grown in wastewater reached an average value of 47%, being statistically different (*p* < 0.05). This result can be justified with the assumption that the biomass, in contact with the wastewater, had a more intense metabolism, with photosynthetic activity in the presence of light and heterotrophic metabolism induced by the presence of organic carbon in the wastewater during the dark hours, consistent with the metabolism of lipids.

Overall, in all the samples was observed an average composition of 45% of carbon and 6% of nitrogen. Selvaratnam, Reddy, Muppaneni, Holguin, Nirmalakhandan, Lammers and Deng [40] estimated a nitrogen content in *G. sulphuraria* in the range 4–8%. For this value, in a hydrothermal liquefaction process such as the POWER system [40] and at the appropriate temperature conditions, with *G. phlegrea* between 55% and 75% of N from the biomass [78] could be easily recovered, thus supporting the optimal recovery of an element which is essential for the production of energy and as a nutrient for new biomass.

As suggested at the beginning of this section, photosynthesis is a process causing depletion of δ^13^C, a trend that is also observable in Figure 6a. In the experimental control the depletion in δ^13^C is weak because A is a growth medium identical to the medium in which the algal stock was usually stored before starting the experiments, with the exception of being free of additional organic carbon, so the algae basically retained an isotopic state of equilibrium with their previous condition. In the case of W, on the contrary, the algae grew in raw wastewater, a matrix generally characterized by a much more negative δ^13^C [79]; since *Galdieria* is able to exploit the organic carbon of the wastewater to practice mixotrophy and heterotrophy, it is reasonable to think that the more intense depletion of δ^13^C observable in W after the first 30′ is associated with the capture of carbon from the growth medium. This is consistent with the observations of Oesterhelt, et al. [80], who demonstrated that *Galdieria* catabolically represses photosynthesis in the presence of organic carbon sources.

With regard to N metabolism, while it does not exhibit major variations in biomass from a quantitative point of view, the time trend of its isotopic ratio (Figure 6b) suggests that *G. phlegrea* grown in wastewater fixed nitrogen from an external source whose δ^15^N had a higher value. Berto, Calace, Rampazzo, Saccomandi and Stellato [77] reported that the range of δ^15^N for raw urban wastewater is between −4/+6. The wastewater used in the experiments was a primary settled wastewater that was mechanically deprived only of suspended solids, oils and sand, but had not yet undergone biological treatments; therefore, it can be assumed that the value of δ^15^N for this liquid phase falls in the range −4/+6. Given this and observing Figure 6b, the trend of δ^15^N in the wastewater can be justified as being related to the assimilation of ammoniacal nitrogen dissolved in the wastewater and enriched in ^15^N, consistently with what was described at the beginning of this section. Moreover, the increase in δ^15^N was statistically significant at all sampling points and, comparing Figure 6b (δ^15^N graph) with Figure 3 (trend of NH_4_^+^), it can be seen that the trend was almost specular to the reduction of NH_4_^+^ in the growth media. Nitrogen uptake is involved in pigment and protein metabolism and algal growth and proliferation. No nitrogen storage granules are known in *Galdieria*, other than the phycobilisome granules located through the thylakoid within the chloroplast, which contain nitrogen. However, in the present work, the level of phycocyanin in *G. phlegrea* grown in wastewater was not significantly different from the control culture. On the other hand, recent genomic analyses revealed that *G. phlegrea* adopted improved strategies for adaptation to specific environments, such as the reacquisition of a complete set of genes required for urea hydrolysis, which is necessary as an alternative source of nitrogen in N-limited environments [81]. Therefore, we can assume that *G. phlegrea* uses N-NH_4_^+^ to convert it to urea as an N-deposit. Concerning the trend of δ^15^N for the biomass grown in Allen, the analysis of the results using Tukey’s range test revealed that there were no statistically significant differences between δ^15^N in Allen at time 0 h and the value assumed by the parameter in the following hours (up to the 24th hour). Hence, it appears that δ^15^N in the autotrophic control (A) substantially retained an equilibrium condition throughout the experiment, in agreement with what was observed for δ^13^C in Allen (Figure 6a).

## 4. Conclusions

Results showed that *G. phlegrea* was able to grow in raw wastewater, where it reached an algal density higher than in the control growth medium. Ammonium and phosphate removal efficiencies were very high, *G. phlegrea* beingable to reduce on average at least 50% of the ammonium and 20% of the phosphate in the wastewater during the first 24 h. Protocols adopted here for biomass pre-treatment and total lipid extraction were more suitable for the characteristics of *Galdieria*, yielding a higher fraction than what the literature reports for species of the same genus. Isotopic analysis helped to reveal carbon and nitrogen pathways and to clearly show that their uptake was related to the medium with which the biomass comes into contact. The results achieved in this laboratory stage encourage further efforts in this direction. Future experiments will perform a scale-up of the process, to simulate a scenario as realistically as possible. High value-added compounds potentially extractable from *G. phlegrea* and the uses of the residual biomass will also be studied in more detail, in order to define the life-cycle benefits of the process and thus validate the potential application of *G. phlegrea* for sustainable wastewater treatment.

## Figures and Tables

**Table 1:** Time sheet of the sampling performed on each of the biological replicates and the analyses that were carried out on each of these samples.

		Sampling Times												
		0 h	0.5 h	3 h	6 h	24 h	Day 2	Day 3	Day 4	Day 5	Day 6	Day 7	Day 8	Day 9
**Monitored parameters**	OD_750_	✔	✔	✔	✔	✔		✔			✔			✔
	Cell counting	✔						✔			✔			✔
	NH_4_^+^	✔	✔	✔	✔	✔	✔	✔						
	PO_4_^3−^	✔	✔	✔	✔	✔								
	Phycocyanin	✔	✔	✔	✔	✔								
	Lipid fraction	✔	✔	✔	✔	✔								
	[C]-[N] fraction	✔	✔	✔	✔	✔								
	δ^13^C-δ^15^N	✔	✔	✔	✔	✔								

## Data Availability

The data presented in this study are available on request from the corresponding author.

## References

[1] Kehrein P., van Loosdrecht M., Osseweijer P., Garfí M., Dewulf J., Posada J. (2020). A critical review of resource recovery from municipal wastewater treatment plants–market supply potentials, technologies and bottlenecks. Environ. Sci. Water Res. Technol..

[2] Capodaglio A.G., Olsson G. (2020). Energy issues in sustainable urban wastewater management: Use, demand reduction and recovery in the urban water cycle. Sustainability.

[3] Li K., Liu Q., Fang F., Luo R., Lu Q., Zhou W., Huo S., Cheng P., Liu J., Addy M. (2019). Microalgae-based wastewater treatment for nutrients recovery: A review. Bioresour. Technol..

[4] Cabanelas I.T.D., Arbib Z., Chinalia F.A., Souza C.O., Perales J.A., Almeida P.F., Druzian J.I., Nascimento I.A. (2013). From waste to energy: Microalgae production in wastewater and glycerol. Appl. Energy.

[5] Jeyanayagam S. (2005). True Confessions of the Biological Nutrient Removal Process. Fla. Water Resour. J..

[6] Clarens A.F., Resurreccion E.P., White M.A., Colosi L.M. (2010). Environmental Life Cycle Comparison of Algae to Other Bioenergy Feedstocks. Environ. Sci. Technol..

[7] Heidrich E.S., Curtis T.P., Dolfing J. (2011). Determination of the internal chemical energy of wastewater. Environ. Sci. Technol..

[8] Tyler H., Fallgren P.H., Song J., Zhiyong Jason R. (2013). Energy and Performance Comparison of Microbial Fuel Cell and Conventional Aeration Treating of Wastewater. J. Microb. Biochem. Technol..

[9] Chrispim M.C., Scholz M., Nolasco M.A. (2019). Phosphorus recovery from municipal wastewater treatment: Critical review of challenges and opportunities for developing countries. J. Environ. Manag..

[10] Ye Y., Ngo H.H., Guo W., Liu Y., Chang S.W., Nguyen D.D., Liang H., Wang J. (2018). A critical review on ammonium recovery from wastewater for sustainable wastewater management. Bioresour. Technol..

[11] Ahn Y.H., Hwang I.S., Min K.S. (2004). ANAMMOX and partial denitritation in anaerobic nitrogen removal from piggery waste. Water Sci. Technol..

[12] Cui Y.-X., Biswal B.K., Guo G., Deng Y.-F., Huang H., Chen G.-H., Wu D. (2019). Biological nitrogen removal from wastewater using sulphur-driven autotrophic denitrification. Appl. Microbiol. Biotechnol..

[13] González I., Herrero N., Siles J.Á., Chica A.F., Martín M., Izquierdo C.G., Gómez J.M. (2020). Wastewater nutrient recovery using twin-layer microalgae technology for biofertilizer production. Water Sci. Technol..

[14] Henkanatte-Gedera S.M., Selvaratnam T., Caskan N., Nirmalakhandan N., Van Voorhies W., Lammers P.J. (2015). Algal-based, single-step treatment of urban wastewaters. Bioresour. Technol..

[15] Johnson D.B., Schideman L.C., Canam T., Hudson R.J.M. (2018). Pilot-scale demonstration of efficient ammonia removal from a high-strength municipal wastewater treatment sidestream by algal-bacterial biofilms affixed to rotating contactors. Algal Res..

[16] Wollmann F., Dietze S., Ackermann J.-U., Bley T., Walther T., Steingroewer J., Krujatz F. (2019). Microalgae wastewater treatment: Biological and technological approaches. Eng. Life Sci..

[17] Goswami R.K., Mehariya S., Verma P., Lavecchia R., Zuorro A. (2020). Microalgae-based biorefineries for sustainable resource recovery from wastewater. J. Water Process. Eng..

[18] Bhatia S.K., Mehariya S., Bhatia R.K., Kumar M., Pugazhendhi A., Awasthi M.K., Atabani A.E., Kumar G., Kim W., Seo S.-O. (2021). Wastewater based microalgal biorefinery for bioenergy production: Progress and challenges. Sci. Total Environ..

[19] Sydney E.B., Schafranski K., Barretti B.R.V., Sydney A.C.N., Zimmerman J.F.D.A., Cerri M.L., Mottin Demiate I. (2019). Biomolecules from extremophile microalgae: From genetics to bioprocessing of a new candidate for large-scale production. Process. Biochem..

[20] Martinez-Garcia M., Kormpa A., van der Maarel M.J.E.C. (2017). The glycogen of *Galdieria sulphuraria* as alternative to starch for the production of slowly digestible and resistant glucose polymers. Carbohydr. Polym..

[21] Carfagna S., Landi V., Coraggio F., Salbitani G., Vona V., Pinto G., Pollio A., Ciniglia C. (2018). Different characteristics of C-phycocyanin (C-PC) in two strains of the extremophilic *Galdieria phlegrea*. Algal Res..

[22] Ferreira G.F., Ríos Pinto L.F., Maciel Filho R., Fregolente L.V. (2019). A review on lipid production from microalgae: Association between cultivation using waste streams and fatty acid profiles. Renew. Sustain. Energy Rev..

[23] Chakraborty M., Miao C., McDonald A., Chen S.L. (2012). Concomitant extraction of bio-oil and value added polysaccharides from Chlorella sorokiniana using a unique sequential hydrothermal extraction technology. Fuel.

[24] Selvaratnam T., Pegallapati A., Montelya F., Rodriguez G., Nirmalakhandan N., Lammers P.J., van Voorhies W. (2015). Feasibility of algal systems for sustainable wastewater treatment. Renew. Energy.

[25] Hena S., Znad H., Heong K., Judd S. (2018). Dairy farm wastewater treatment and lipid accumulation by Arthrospira platensis. Water Res..

[26] Moreno Osorio J.H., Luongo V., Del Mondo A., Pinto G., Pollio A., Frunzo L., Lens P.N.L., Esposito G. (2018). Nutrient removal from high strength nitrate containing industrial wastewater using Chlorella sp. strain ACUF_802. Ann. Microbiol..

[27] Martínez M.E., Sánchez S., Jiménez J.M., El Yousfi F., Muñoz L. (2000). Nitrogen and phosphorus removal from urban wastewater by the microalga Scenedesmus obliquus. Bioresour. Technol..

[28] Zhai J., Li X., Li W., Rahaman M.H., Zhao Y., Wei B., Wei H. (2017). Optimization of biomass production and nutrients removal by Spirulina platensis from municipal wastewater. Ecol. Eng..

[29] Čížková M., Vítová M., Zachleder V., Vítová M. (2019). The Red Microalga *Galdieria* as a Promising Organism for Applications in Biotechnology. Biotechnology, Microalgae–From Physiology to Application.

[30] Biswal B.K., Mazza A., Masson L., Gehr R., Frigon D. (2014). Impact of wastewater treatment processes on antimicrobial resistance genes and their co-occurrence with virulence genes in Escherichia coli. Water Res..

[31] Cho C.H., Park S.I., Ciniglia C., Yang E.C., Graf L., Bhattacharya D., Yoon H.S. (2020). Potential causes and consequences of rapid mitochondrial genome evolution in thermoacidophilic *Galdieria* (Rhodophyta). BMC Evol. Biol..

[32] Ciniglia C., Cennamo P., De Natale A., De Stefano M., Sirakov M., Iovinella M., Yoon H.S., Pollio A. (2019). Cyanidium chilense (Cyanidiophyceae, Rhodophyta) from tuff rocks of the archeological site of Cuma, Italy. Phycol. Res..

[33] Eren A., Iovinella M., Yoon H.S., Cennamo P., de Stefano M., de Castro O., Ciniglia C. (2018). Genetic structure of *Galdieria* populations from Iceland. Polar Biol..

[34] Ciniglia C., Yoon H.S., Pollio A., Pinto G., Bhattacharya D. (2004). Hidden biodiversity of the extremophilic Cyanidiales red algae. Mol. Ecol..

[35] Iovinella M., Carbone D.A., Diana C., Seth J.D., Michele I., Esposito S., Ciniglia C. (2020). Prevalent pH Controls the Capacity of *Galdieria* maxima to Use Ammonia and Nitrate as a Nitrogen Source. Plants.

[36] Salbitani G., Cipolletta S., Vona V., Di Martino C., Carfagna S. (2020). Heterotrophic Cultures of *Galdieria phlegrea* Shift to Autotrophy in the Presence or Absence of Glycerol. J. Plant. Growth Regul..

[37] Minoda A., Sawada H., Suzuki S., Miyashita S., Inagaki K., Yamamoto T., Tsuzuki M. (2015). Recovery of rare earth elements from the sulfothermophilic red alga *Galdieria sulphuraria* using aqueous acid. Appl. Microbiol. Biotechnol..

[38] Selvaratnam T., Pegallapati A.K., Reddy H., Kanapathipillai N., Nirmalakhandan N., Deng S., Lammers P.J. (2015). Algal biofuels from urban wastewaters: Maximizing biomass yield using nutrients recycled from hydrothermal processing of biomass. Bioresour. Technol..

[39] Tchinda D., Henkanatte-Gedera S.M., Abeysiriwardana-Arachchige I.S.A., Delanka-Pedige H.M.K., Munasinghe-Arachchige S.P., Zhang Y., Nirmalakhandan N. (2019). Single-step treatment of primary effluent by *Galdieria sulphuraria*: Removal of biochemical oxygen demand, nutrients, and pathogens. Algal Res..

[40] Selvaratnam T., Reddy H., Muppaneni T., Holguin F.O., Nirmalakhandan N., Lammers P.J., Deng S. (2015). Optimizing energy yields from nutrient recycling using sequential hydrothermal liquefaction with *Galdieria sulphuraria*. Algal Res..

[41] Henkanatte-Gedera S.M., Selvaratnam T., Karbakhshravari M., Myint M., Nirmalakhandan N., Van Voorhies W., Lammers P.J. (2017). Removal of dissolved organic carbon and nutrients from urban wastewaters by *Galdieria sulphuraria*: Laboratory to field scale demonstration. Algal Res..

[42] Cheng F., Jarvis J.M., Yu J., Jena U., Nirmalakhandan N., Schaub T.M., Brewer C.E. (2019). Bio-crude oil from hydrothermal liquefaction of wastewater microalgae in a pilot-scale continuous flow reactor. Bioresour. Technol..

[43] Li Y., Slouka S.A., Henkanatte-Gedera S.M., Nirmalakhandan N., Strathmann T.J. (2019). Seasonal treatment and economic evaluation of an algal wastewater system for energy and nutrient recovery. Environ. Sci. Water Res. Technol..

[44] Selvaratnam T., Pegallapati A.K., Montelya F., Rodriguez G., Nirmalakhandan N., Van Voorhies W., Lammers P.J. (2014). Evaluation of a thermo-tolerant acidophilic alga, *Galdieria sulphuraria*, for nutrient removal from urban wastewaters. Bioresour. Technol..

[45] Pinto G., Ciniglia C., Cascone C., Pollio A., Seckbach J. (2007). Species Composition of Cyanidiales Assemblages in Pisciarelli (Campi Flegrei, Italy) and Description of *Galdieria Phlegrea* SP. NOV. Algae and Cyanobacteria in Extreme Environments.

[46] Barcytė D., Elster J., Nedbalová L. (2018). Plastid-encoded rbcL phylogeny suggests widespread distribution of *Galdieria phlegrea* (Cyanidiophyceae, Rhodophyta). Nord. J. Bot..

[47] Di Cicco M.R., Spagnuolo A., Masiello A., Vetromile C., Nappa M., Corbo G., Lubritto C. (2019). Energy Monitoring of a Wastewater Treatment Plant in Salerno, Campania Region (Southern Italy). Frontiers in Water-Energy-Nexus—Nature-Based Solutions, Advanced Technologies and Best Practices for Environmental Sustainability, Proceedings of the 2nd WaterEnergyNEXUS Conference, 14–17 November 2018, Salerno, Italy.

[48] Di Cicco M.R., Spagnuolo A., Masiello A., Vetromile C., Nappa M., Corbo G., Lubritto C. (2019). Assessing energy performance and critical issues of a large wastewater treatment plant through full-scale data Benchmarking. Water Sci. Technol..

[49] Di Cicco M.R., Spagnuolo A., Masiello A., Vetromile C., Nappa M., Lubritto C. (2020). Energetic and environmental analysis of a wastewater treatment plant through static and dynamic monitoring activities. Int. J. Environ. Sci. Technol..

[50] Allen M.B. (1959). Studies with cyanidium caldarium, an anomalously pigmented chlorophyte. Arch. Mikrobiol..

[51] LeGresley M., McDermott G. (2010). Counting chamber methods for quantitative phytoplankton analysis—haemocytometer, Palmer-Maloney cell and Sedgewick-Rafter cell. UNESCO (IOC Man. Guides).

[52] Hernández-López J., Vargas-Albores F. (2003). A microplate technique to quantify nutrients (NO₂^−^, NO₃^−^,NH₄⁺ and PO₄^3−^) in seawater. Aquac. Res..

[53] Silveira S.T., Burkert J.F., Costa J.A., Burkert C.A., Kalil S.J. (2007). Optimization of phycocyanin extraction from Spirulina platensis using factorial design. Bioresour. Technol..

[54] Rahman D.Y., Sarian F.D., van Wijk A., Martinez-Garcia M., van der Maarel M. (2017). Thermostable phycocyanin from the red microalga Cyanidioschyzon merolae, a new natural blue food colorant. J. Appl. Phycol..

[55] Onay M., Sonmez C., Oktem H.A., Yucel M. (2016). Evaluation of various extraction techniques for efficient lipid recovery from thermo-resistant microalgae, Hindakia, Scenedesmus and Micractinium Species. Am. J. Anal. Chem..

[56] Scirè-Calabrisotto C., Webb J.M., Frankel D., Ricci P., Altieri S., Lubritto C. (2020). New evidence for diet and subsistence economy in Early and Middle Bronze Age Cyprus. J. Archaeol. Sci. Rep..

[57] Tiwari M., Singh A.K., Sinha D.K., Ramkumar M. (2015). Chapter 3–Stable Isotopes: Tools for Understanding Past Climatic Conditions and Their Applications in Chemostratigraphy. Chemostratigraphy.

[58] Kounaves S.P., Oberlin E.A., Filiberto J., Schwenzer S.P. (2019). Chapter 9–Volatiles Measured by the Phoenix Lander at the Northern Plains of Mars. Volatiles in the Martian Crust.

[59] Condie K.C., Condie K.C. (2005). 7–Living Systems. Earth as an Evolving Planetary System.

[60] Coplen T.B., Brand W.A., Gehre M., Gröning M., Meijer H.A.J., Toman B., Verkouteren R.M. (2006). New Guidelines for δ13C Measurements. Anal. Chem..

[61] Bohlke J.K., Gwinn C.J., Coplen T.B. (1993). New reference materials for nitrogen isotope ratio measurements. Geostand. Newsl..

[62] Fuggi A., Di Martino Rigano V., Vona V., Rigano C. (1981). Nitrate and ammonium assimilation in algal cell-suspensions and related pH variations in the external medium, monitored by electrodes. Plant. Sci. Lett..

[63] Fuggi A., Rigano V.D.M., Vona V., Rigano C. (1981). Pattern of inhibition of nitrate utilization by ammonium in the acidophilic thermophilic unicellular alga Cyanidium caldarium. Arch. Microbiol..

[64] Rigano C., Di Martino Rigano V., Vona V., Fuggi A. (1981). Nitrate reductase and glutamine synthetase activities, nitrate and ammonia assimilation, in the unicellular alga Cyanidium caldarium. Arch. Microbiol..

[65] Woertz I., Feffer A., Lundquist T., Nelson Y. (2009). Algae Grown on Dairy and Municipal Wastewater for Simultaneous Nutrient Removal and Lipid Production for Biofuel Feedstock. J. Environ. Eng..

[66] Su Y., Mennerich A., Urban B. (2011). Municipal wastewater treatment and biomass accumulation with a wastewater-born and settleable algal-bacterial culture. Water Res..

[67] Hu B., Min M., Zhou W., Li Y., Mohr M., Cheng Y., Lei H., Liu Y., Lin X., Chen P. (2012). Influence of Exogenous CO_2_ on Biomass and Lipid Accumulation of Microalgae Auxenochlorella protothecoides Cultivated in Concentrated Municipal Wastewater. Appl. Biochem. Biotechnol..

[68] Samorì G., Samorì C., Guerrini F., Pistocchi R. (2013). Growth and nitrogen removal capacity of Desmodesmus communis and of a natural microalgae consortium in a batch culture system in view of urban wastewater treatment: Part I. Water Res..

[69] Alishah Aratboni H., Rafiei N., Garcia-Granados R., Alemzadeh A., Morones-Ramírez J.R. (2019). Biomass and lipid induction strategies in microalgae for biofuel production and other applications. Microb. Cell Factories.

[70] Zhu L.D., Li Z.H., Hiltunen E. (2016). Strategies for Lipid Production Improvement in Microalgae as a Biodiesel Feedstock. Biomed Res. Int..

[71] Graziani G., Schiavo S., Nicolai M.A., Buono S., Fogliano V., Pinto G., Pollio A. (2013). Microalgae as human food: Chemical and nutritional characteristics of the thermo-acidophilic microalga *Galdieria sulphuraria*. Food Funct..

[72] López G., Yate C., Ramos F.A., Cala M.P., Restrepo S., Baena S. (2019). Production of Polyunsaturated Fatty Acids and Lipids from Autotrophic, Mixotrophic and Heterotrophic cultivation of *Galdieria* sp. strain USBA-GBX-832. Sci. Rep..

[73] Bligh E.G., Dyer W.J. (1959). A rapid method of total lipid extraction and purification. Can. J. Biochem. Physiol..

[74] Sakurai T., Aoki M., Ju X., Ueda T., Nakamura Y., Fujiwara S., Umemura T., Tsuzuki M., Minoda A. (2016). Profiling of lipid and glycogen accumulations under different growth conditions in the sulfothermophilic red alga *Galdieria sulphuraria*. Bioresour. Technol..

[75] Minoda A., Sato N., Nozaki H., Okada K., Takahashi H., Sonoike K., Tsuzuki M. (2002). Role of sulfoquinovosyl diacylglycerol for the maintenance of photosystem II in Chlamydomonas reinhardtii. Eur. J. Biochem..

[76] Weber A.P.M., Horst R.J., Barbier G.G., Oesterhelt C. (2007). Metabolism and Metabolomics of Eukaryotes Living Under Extreme Conditions. International Review of Cytology.

[77] Berto D., Calace N., Rampazzo F., Saccomandi F., Stellato L. (2018). Isotopi: Dalla teoria alla pratica. Transl. “Isotopes: From theory to practice”.

[78] Yu G., Zhang Y., Schideman L., Funk T., Wang Z. (2011). Distributions of carbon and nitrogen in the products from hydrothermal liquefaction of low-lipid microalgae. Energy Environ. Sci..

[79] Toyoda S., Suzuki Y., Hattori S., Yamada K., Fujii A., Yoshida N., Kouno R., Murayama K., Shiomi H. (2011). Isotopomer Analysis of Production and Consumption Mechanisms of N2O and CH4 in an Advanced Wastewater Treatment System. Environ. Sci. Technol..

[80] Oesterhelt C., Schmalzlin E., Schmitt J.M., Lokstein H. (2007). Regulation of photosynthesis in the unicellular acidophilic red alga *Galdieria sulphuraria*. Plant. J..

[81] Qiu H., Price D.C., Weber A.P.M., Reeb V., Chan Yang E., Lee J.M., Kim S.Y., Yoon H.S., Bhattacharya D. (2013). Adaptation through horizontal gene transfer in the cryptoendolithic red alga *Galdieria phlegrea*. Curr. Biol..

